# Effect and safety of acupuncture for autism spectrum disorders: A protocol for systematic review and meta-analysis

**DOI:** 10.1097/MD.0000000000022269

**Published:** 2021-03-19

**Authors:** Ying Zhang, Jianfei Zeng, Dan Wu, Xiujuan Li, Yangxia Chen, Shixia Dai, Bing Wang, Yu Qi, Jianping Lu

**Affiliations:** a Department of Child Psychiatry; b Shenzhen Kangning Hospital and Shenzhen Mental Health Center, Shenzhen, Guangdong Province, China.

**Keywords:** acupuncture, autism spectrum disorders, meta-analysis, protocol

## Abstract

**Background:**

Autism spectrum disorder (ASD) is a complex neurodevelopmental condition, which is characterized by impairment in social interaction or communication and lack of flexibility of imagination and behavior. Acupuncture is one of the most common modality of Traditional Chinese medicine (TCM) and has been used to treat various disease in clinical practice for more than 2000 years in China by correcting disharmony and dysregulation of body. It has sometimes been used as a treatment aimed at improving ASD symptoms and outcomes, but its clinical effectiveness and safety has not been rigorously reviewed. We will plan to conduct a systematic review and meta-analysis to summarize the current evidence on the effects and safety of acupuncture for ASD.

**Methods:**

The following databases will be searched: PubMed, the Cochrane Library, Embase, Wanfang Data, China National Knowledge Infrastructure, SinoMed, and VIP. Randomised controlled trials will be included to evaluate the effect and safety of acupuncture on patients with ASD. The primary outcome will be the core features of ASD. The risk of bias will be assessed by the Cochrane risk of bias tool. We will conduct a meta-analysis and sensitivity analysis, as well as a subgroup analysis if high heterogeneity is present, using Revman 5.3. We will use funnel plots to identify potential reporting biases. The Grading of Recommendations Assessment, Development and Evaluation will be used to evaluate the quality of evidence.

**Results:**

This study will be to assess the effect and safety of acupuncture for ASD.

**Conclusions:**

This study will assess the effect of acupuncture for ASD and provide reliable evidence for the choice of treatments.

**Ethics and dissemination:**

The protocol will not need ethical approval because no issues of participant privacy exist. The results of this systematic review will provide evidence about the effect and safety of acupuncture for ASD. The results will be disseminated through peer review.

## 1 Introduction

Autism spectrum disorder (ASD) is a complex neurodevelopmental condition, which is characterized by impairment in social interaction or communication and lack of flexibility of imagination and behavior.^[1]^ Nowadays, the etiology and pathogenesis of ASD remains to be seen. Nevertheless, a number of studies frames ASD not as a single disease but as a combination of heredity and environment.^[2–9]^ In mainland China, the prevalence of ASD is 39.23 per 10,000^[10]^ versus 34 per 10,000 in USA.^[11]^ In more recent studies, ASD does not only have an increasing trend, but also brings heavy burden of care to families and society.

Acupuncture is one of the most common modality of Traditional Chinese medicine(TCM) and has been used to treat various disease in clinical practice for more than 2000 years in China by correcting disharmony and dysregulation of body.^[12]^ Acupuncture is widely used in ASD in China as an effective treatment approach. Its mechanism may be to modulate neuroendocrine system, such as neurotransmitters, neurotrophins, neuroinflammation and apoptosis of neural tissues.

Up to present, some studies indicated that acupuncture could improve clinical symptoms of children and adults ASD.^[13–15]^ However, there were some disadvantages in these studies, including small sample size, unclear or inadequate study design, et al So we aim to summarize the up-to-date information on acupuncture for autism spectrum disorder to evaluate its effect and safety in clinical practice.

### 1.1 Objectives and Registration

We will summarize the evidence to evaluate the effect and safety of acupuncture for autism spectrum disorders. Based on the Preferred Reporting Items for Systematic Reviews and Meta-Analyses Statement,^[16]^ this review protocol is registered in the OSF platform (https://osf.io/registries) with a registration number 10.17605/OSF.IO/JZA4Y.

### 1.2 Inclusion criteria

#### 1.2.1 Types of studies

We will include all randomized controlled trials that assessed the effect and safety of acupuncture for the participants with ASD in this systematic review regardless of publication status and language.

#### 1.2.2 Types of participants

We will include all participants diagnosed as ASD regardless of their age, sex, or race.

#### 1.2.3 Types of intervention

We will include all forms of acupuncture interventions including needling, acupressure, electroacupuncture, fire needle, auriculotherapy, laser acupuncture, scalp acupuncture, hand acupuncture, and bee venom acupuncture. The control interventions will be no treatment, placebo or sham acupuncture, and therapeutic agents.

#### 1.2.4 Types of outcome measures

Primary outcomes will be the core features of ASD, including impairments on social interaction or communication and defect of imagination and behavior by effective measure tools. Secondary outcomes will be quality of life and adverse events.

### 1.3 Search methods for the identification of studies

We will retrieve Cochrane Library, MEDLINE, EmBase, Chinese BioMedical Database, China National Knowledge Infrastructure, Chinese VIP Information (VIP) and Wangfang Database regardless of publication date or language. We will conduct different strategies for different electronic databases based on disease terms (autism spectrum disorders, pervasive developmental disorder, and Asperger), intervention terms (acupuncture, electroacupuncture and acupoints).

### 1.4 Data collection

#### 1.4.1 Selection of studies

**Figure 1:**
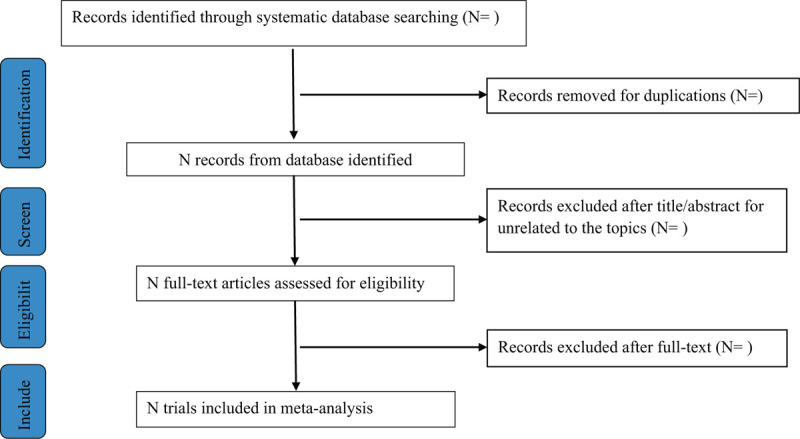
Flow chart of study selection.

We will retrieve and manage all potentially eligible articles in the Mendeley reference manager software and duplicate publications will be deleted. Two review authors (ZY and ZJF) will independently scan the titles and abstracts of all potentially eligible articles identified from electronic databases. Full-text articles will be scanned for all potentially relevant articles. We will discuss any disagreement on the selection of articles with arbiter (LJP). The Preferred Reporting Items for Systematic Reviews and Meta-Analyses flow chart is displayed in Figure 1.

#### 1.4.2 Data extraction and management

Two review authors (ZY and ZJF) will independently extract the relevant information based on a standard data extraction table. Information will include publication of year, author, participants, intervention, control, duration of intervention, outcomes and methodological characteristics. We will resolve any disagreement on data extraction with the arbiter (LJP).

### 1.5 Assessment of the risk of bias

Two authors (ZY and ZJF) will independently assess the risk of bias using the Cochrane Collaborations tool. Six potential items will be assessed: sequence generation, allocation concealment, blinding, incomplete outcomes, selective reporting and other bias. The judgments of evaluated domains will include low, unclear and high. We will resolve the disagreement on assessment of bias with the arbiter (LJP).

### 1.6 Assessment of reporting biases

We will explore the potential publication bias by funnel plots if sufficient studies were included. Asymmetry of funnel plots will suggest possible small study effects and we will explain the results cautiously.^[17,18]^

### 1.7 Assessment of heterogeneity

We will examine heterogeneity for quantifying inconsistency by the *I*^2^ statistic in all included studies. If *I*^2^ value > 50%, substantial heterogeneity will be indicated.

### 1.8 Data synthesis and statistical analysis

We will perform all the data analysis by Review Manager software 5.3 based on intent-to-treat (ITT) principle. Discontinuous variables will be summarized as risk ratios with their 95% confidence intervals and continuous variables will be expressed as mean differences with their 95% confidence intervals. We will evaluate the heterogeneity for quantifying inconsistency in the included studies by Higgins *I*^2^ statistic. Standard meta-analysis in random effects model will be conducted if *I*^2^ >0.5. If not, the fixed effect model will perform. For missing data, we will contact the authors by e-mail as much as possible.

#### 1.8.1 Subgroup and sensitivity analysis

We will conduct subgroup analysis to explore the differences in the type of participants, sex, methodological quality and race/ethnicity if there is significant heterogeneity. To assess the robustness of the data synthesis, we will perform sensitivity analysis if possible.

#### 1.8.2 Confidence in cumulative evidence

We will assess the level of evidence on venous thrombosis by Grading of Recommendations Assessment, Development and Evaluation.^[19]^ The quality of the body of evidence will be assessed based on 5 factors, including study limitations, effect consistency, imprecision, indirectness, and publication bias. The assessments will be categorized as high, moderate, low, and very low quality.

## 2 Ethics and dissemination

Ethical approval is not appropriate, on account of this protocol for systematic review and meta-analysis. In our study there will be no patients recruited, and no data gathered from patients. This review will be disseminated by the approach of peer-reviewed publications. Methods and analysis

## Author contributions

LJP and ZY developed the study protocol. ZY and ZJF developed the search strategy. ZY and ZJF will scan the included studies, extract the data and assess the risk of bias with supervision of LJP. ZY and ZJF will perform data analysis. All authors (ZY, ZJF, WD, LXJ, CYX, DSX, WB, QY, and LJP) will contribute to data interpretation. ZY and LJP drafted and revised the manuscript. All authors have read and approved the final version of the manuscript.

**Conceptualization:** Ying Zhang, Jian-Ping Lu.

**Data curation:** Ying Zhang, Jian-Fei Zeng.

**Formal analysis:** Ying Zhang, Jian-Fei Zeng.

**Funding acquisition:** Jian-Ping Lu.

**Methodology:** Ying Zhang, Jian-Fei Zeng, Jian-Ping Lu.

**Software:** Ying Zhang, Jian-Fei Zeng.

**Supervision:** Jian-Ping Lu.

**Validation:** Ying Zhang, Dan Wu, Xiu-Juan Li, Yang-Xia Chen, Shi-xia Dai, Bing Wang, Yu Qi, Jian-Ping Lu.

**Writing – original draft:** Ying Zhang, Yu Qi, Jian-Ping Lu.

**Writing – review & editing:** Ying Zhang, Jian-Fei Zeng, Dan Wu, Xiu-Juan Li, Yang-Xia Chen, Shi-xia Dai, Bing Wang, Yu Qi, Jian-Ping Lu.
